# *intI*1 gene abundance from septic tanks in Thailand using validated *intI*1 primers

**DOI:** 10.1128/aem.01071-23

**Published:** 2023-10-24

**Authors:** Valentine Okonkwo, Fabien Cholet, Umer Z. Ijaz, Thammarat Koottatep, Tatchai Pussayanavin, Chongrak Polpraset, William T. Sloan, Stephanie Connelly, Cindy J. Smith

**Affiliations:** 1 Department of Infrastructure and Environment, James Watt School of Engineering, University of Glasgow, Glasgow, United Kingdom; 2 School of Environment, Resources and Development, Asian Institute of Technology, Khlong Nueng, Thailand; 3 Faculty of Science, Ramkhamhaeng University, Bangkok, Thailand; 4 Thammasat School of Engineering, Thammasat University, Bangkok, Thailand; Georgia Institute of Technology, Atlanta, Georgia, USA

**Keywords:** wastewater treatment, septic tanks, Thailand, AMR, *intI*1, class 1 integron integrase, *intI*1 qPCR primers, ARGs, decentralized wastewater treatment

## Abstract

Antimicrobial resistance (AMR) poses a serious global health threat, and wastewater treatment (WWT), including septic tanks, is a source of AMR. In Thailand, antibiotics are unregulated, and septic tanks are commonly used. Yet, their impact on the spread or mitigation of AMR is unknown. We monitored household and healthcare conventional septic tanks (CST) and household solar septic tanks (SST) in Thailand using the class 1 integron-integrase (*intI*1) gene abundance as a proxy for AMR. A systematic review of the literature found 65 *intI*1 primers. We evaluated the coverage and specificity of each, including a new MGB TaqMan primer-probe, against clinical and environmental *intI*1, *intI*1-like, and non-*intI*1 databases. The three best primers were selected, laboratory validated for DNA and mRNA quantification, and used to quantify septic tank *intI*1 gene abundance. No primer set could distinguish between *intI*1 and *intI*1-like sequences. While primer choice did not affect gene abundance of the same sample (*P*-value > 0.05), sometimes when comparing the same samples quantified by different primers, statistical differences were observed for one but not the other primer set. This may lead to different interpretations of AMR risk. Irrespective of primers or reactor type *intI*1 gene abundance was greatest in influent > effluent > sludge. *intI*1 gene abundance was lowest in the effluent of the SST-household < CST-household < CST-healthcare. 31% to 42% of *intI*1 was removed by the CST-household tank, indicating while septic tanks remove some *intI*1 they remain a source to the surrounding environment. Toward the goal of achieving standardization across studies, we recommend the F3-R3 primer for *intI*1 quantification.

**IMPORTANCE:**

Antimicrobial resistance is a global crisis, and wastewater treatment, including septic tanks, remains an important source of antimicrobial resistance (AMR) genes. The role of septic tanks in disseminating class 1 integron, and by extension AMR genes, in Thailand, where antibiotic use is unregulated remains understudied. We aimed to monitor gene abundance as a proxy to infer potential AMR from septic tanks in Thailand. We evaluated published *intI*1 primers due to the lack of consensus on optimal Q-PCR primers and the absence of standardization. Our findings confirmed septic tanks are a source of class 1 integron to the environment. We highlighted the significance of *intI*1 primer choice, in the context of interpretation of risk associated with AMR spread from septic tanks. We recommend the validated set (F3-R3) for optimal *intI*1 quantification toward the goal of achieving standardization across studies.

## INTRODUCTION

Antimicrobial resistance (AMR), the ability of microbes to grow and thrive in the presence of compounds capable of limiting their cellular growth or killing cells, is a serious growing public health concern globally; and it has recently been classified as “one of the top ten global threats facing humanity” by the World Health Organization (1).

The occurrence of AMR *via* mutation and subsequent vertical gene transfer or acquisition of AMR genes *via* horizontal gene transfer (HGT) is an inevitable natural phenomenon in the evolution of microbes (2, 3). Nonetheless, recent global challenges including extensive consumption and misuse of antimicrobials, particularly antibiotics, in clinical settings, agri-and aqua-culture, and their subsequent release to the environment, have given rise to the emergence and rapid dissemination of AMR genes among bacteria, including microbes of clinical importance, and the environment (2). Consequently, high global mortality, as a result of patient treatment failure, has been associated with AMR-related infections (700,000 deaths in 2014) (4). Moreover, the global death toll from AMR-related infections has been projected to increase to 10 million deaths per year by 2050 surpassing death from cancer, assuming no change to the current trends/policies, coupled with an economic burden of 100 trillion US Dollars (4).

Wastewater treatment (WWT), including decentralized treatment systems such as septic tanks, receives significant amounts of antibiotics from human and animal waste (30%–90% of antibiotics are excreted in urine and feces (5)) and are now recognized as important reservoirs for AMR creating hotspots for transfer and subsequent release to the environment (3, 6, 7). The selective pressure introduced by the often multiple, low-level, sub-inhibitory concentrations of antimicrobials found in wastewater (WW), promotes AMR gene acquisition among microbes *via* HGT and selection for AMR bacteria. WWT and septic tanks are unable to effectively remove these (3, 8, 9), resulting in increased AMR genes and bacteria discharged directly to the environment, contributing significantly to the global burden (10). The global AMR burden from wastewater is further exacerbated in the Global South due to the high prevalence of extensive antibiotic usage-propelled by poor regulations on usage, ineffective or lacking WWT, coupled with increasing populations and rapidly expanding megacities.

The necessity to tackle AMR discharge from WWT to the environment requires a comprehensive understanding of the role of WWT in the dissemination of AMR to the environment. This understanding will create unique opportunities to implement key strategies to mitigate AMR spread, and, in turn, allow for the safeguarding of global public health. This knowledge can be informed by sensitive, accurate detection, quantification, and tracking of AMR genes from the source (e.g., WWT) to the environment. However, multiple AMR genes exist within WWT. Monitoring numerous AMR genes simultaneously is a major challenge (8), particularly if a rapid assessment is needed. Similarly, monitoring one or a subset of AMR genes is not ideal, as selected AMR gene(s) may be absent (8). Previously, the clinical class 1 integron (CL1-integron) integrase gene (*intI*1) was proposed as a proxy for inferring potential AMR, which circumvents multiple monitoring limitations, by acting as a proxy for potential AMR pollution (8). *intI*1 was proposed as a proxy because it is linked to genes that confer resistance to antibiotics, disinfectants, and heavy metals; it is found in diverse taxonomic groups of pathogenic and non-pathogenic bacteria and can move across taxa *via* HGT due to its physical linkage to mobile genetic elements (MGEs) such as plasmid and transposons; its abundance can rapidly change in response to external pressures such as the presence of antibiotics; selection pressures imposed by recent human activities have resulted in the emergence of the highly conserved clinical *intI*1 variant (8); and the elevated presence of which in the environment indicates pollution and potential hotspot for AMR transfer (8, 11).

Currently, molecular approaches, specifically real-time quantitative PCR (Q-PCR), have emerged as the methods of choice for AMR and CL1-integron detection and quantification in the environment. By far, the most prevalent approach for detecting or quantifying the CL1-integron is the amplification of the *intI*1 gene at the 5′ conserved segment (CS) across diverse ecological niches including engineered systems, for example, WWTs (12 – 14) and natural ecosystems such as sediments (15, 16). While targeting the *intI*1 gene provides no information about the structure beyond the 5′ CS (17), quantification of the *intI*1 gene as an initial screening to infer potential AMR contamination within complex environments is invaluable and a useful initial screening approach. However, within the literature, numerous primers targeting the *intI*1 gene are available (see Table S1) and different sets are used across different studies. The current lack of standardization prevents cross-study comparisons and limits the current understanding of AMR in the environment. As such, selecting optimal *intI*1 primers with both high coverage and specificity suitable for environmental monitoring is a challenge. Moreover, several primers have been designed based on the highly conserved clinical *intI*1 gene sequences (≥98% protein similarity to each other), and the extent to which these primers target the less conserved *intI*1 gene variants (<98% protein similarity) found also in environmental samples (8, 17 – 19) and on the chromosome non-pathogenic *Betaproteobacteria* which carries gene cassettes not associated with AMR genes (19) has yet to be determined. As such, a comprehensive and comparative evaluation of published *intI*1 primers to determine their coverage and specificity against clinical and environmental *intI*1 sequences to identify a consensus optimal *intI*1 primers for monitoring AMR within environmental samples is urgently needed (20).

With this need identified, we undertook to review, evaluate, and then apply *intI*1 primers to quantify the gene across a suite of wastewater samples from septic tanks in Thailand. Specifically, we compare the recent solar septic tank (SST) technology currently implemented in some areas of Thailand (7, 21) to that of conventional septic tanks (CST) treating household and healthcare wastewater. The SST technology differs from CST primarily by the incorporation of a central disinfection chamber containing a heated copper coil connected to a passive solar heat collection system installed on the roof of the toilet block served by the SST (7, 21). The heat from the central chamber (50°C–60°C by design) promotes partial pasteurization as the effluent passes through the chamber prior to discharge. Effluent water quality is improved by reducing microbial biomass including potential pathogens, and by extension reduction in the microbial load should reduce the AMR burden on receiving water bodies. Moreover, the in-tank temperature is raised by the passive transfer of heat from the chamber to the rest of the tank, thus promoting enhanced microbial degradation of both retained solids (sludge) and soluble compounds (7). As such, we hypothesize that *intI*1gene abundance would be lower in the SST than in the CST sludge and effluent owing to the enhanced treatment caused by the increased temperature.

To address this methodological knowledge gap and our hypothesis, a systematic review of the literature was undertaken to obtain published *intI*1 primers followed by a comprehensive *in silico* analysis of primer coverage and specificity against a curated database of clinical and environmental *intI*1 sequences to select the best-performing primers. A subset of the best-performing primer sets was used to quantify the *intI*1 gene abundance from 30 septic tank wastewater samples comparing conventional (healthcare and household wastewater) and solar septic tank (household wastewater), with *intI*1 specificity validated by Illumina MiSeq. We further confirmed the suitability of the primers to quantify *intI*1 gene transcripts. Thus, we propose a validated *intI*1 primer set for quantification of genes and transcripts from environmental samples toward the goal of achieving standardization across *intI*1 studies.

## MATERIALS AND METHODS

### *Inti*1 primer evaluation

#### A systematic review of literature and alignment of primers to *intI*1 reference sequence

A systematic review of >3,000 peer-reviewed publications was conducted to retrieve *intI*1 primer and probe sequences across a range of settings including clinical and environmental, for example, agricultural and human-impacted settings including WWTPs. For this, the “Web of Knowledge” database (https://www.webofscience.com/; last assessed 04/10/2022) was searched using the term “Class 1 integron.” Only published articles in the English language were considered. In all, 3,266 published articles were subsequently recovered. The *intI*1 primer sequences from the respective literature were either retrieved in the main text or from the accompanying supplementary material.

Obtained *intI*1 primer and probe sequences were aligned to a reference *Pseudomonas aeruginosa* plasmid pVS1 nucleotide sequence (M73819.1) using the ClustalX2 algorithm (version 2.1.0.0), with default settings (22) and visualized with BioEdit (version 7.0.5.3) (23). The alignment position of each primer and probe sequence was renamed according to position along the *P. aeruginosa* reference *intI*1 gene sequence (Fig. S1; Table S1).

#### Database construction and curation

**Fig 1:**
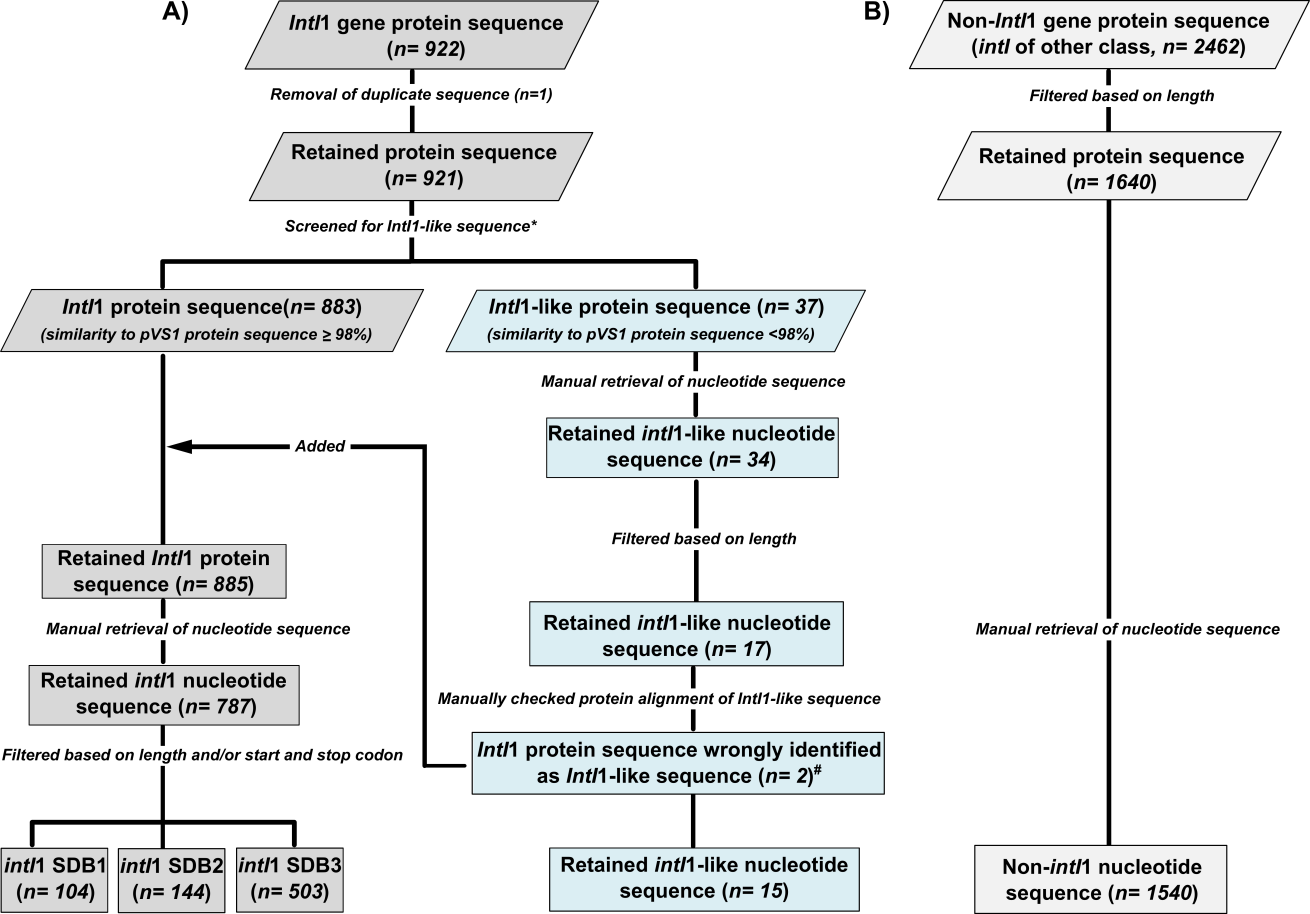
Workflow of integrase sub-databases construction for primer evaluation from 922 *IntI*1 (A) and 2,462 non-*IntI*1 protein sequences (B). Duplicate *IntI*1 protein sequence (*n = 1*) was discarded. Retained protein sequences were compared to the reference *IntI*1 protein of pVS1 plasmid (AAA25857.1) using NCBI BlastP to identify true *intI*1 sequences. *IntI*1 protein sequences with a ≥98% identity to the pVS1 protein sequence were classified as *IntI*1 protein sequences. Conversely, *IntI*1 protein sequences with a <98% identity to the pVS1 protein sequence were classified as *IntI*1-like sequences. Three *intI*1 gene nucleotide sub-databases (SDB1, SDB2, and SDB3) were finally constructed based on criteria specified in Table 1 and were used to evaluate the coverage of primers. *intI*1-Like (*n = 15*) and non-*intI*1 (*n = 1540*) sub-databases were used to evaluate the specificity of primers. * Indicates removal of 1 protein sequence (CP006631.1) from the 921 non-duplicate *intI*1 protein sequence, due to no similarity score to the *IntI*1 pVS1 protein sequence generated, as a result of low sequence similarity. ^#^ Indicates the two (WP_058137959.1 and WP_058135314.1) *IntI*1 protein sequences incorrectly identified as *IntI*1-like protein sequences by the low similarity score generated by NCBI following alignment to the pSV1 protein sequence due to these sequences being partial length sequences.

The integron-integrase database by Zhang et al. (20), consisting of 922 and 2,462 *intI*1 gene and integron-integrase (*intI*) of other class protein sequences, respectively (herein referred to as non-*intI*1 database) (Fig. 1), was employed for the analysis of primers. While the *intI* of other class databases was mostly populated with protein sequences from other integron-integrase classes, it also contained a number of XerCs integrases (*n = 78*) and transposases protein sequences (*n = 66*) as recently reported by Roy and co-workers (24). In this study, however, the inclusion of these protein sequences within the non-*intI*1 database is not of significance, as the goal was to confirm that analyzed *intI*1 primer sets were unable to amplify sequences within this database *via in silico* testing, thus confirming their specificity.

**TABLE 1:** Criterion for construction of integrase sub-databases

Sub_databases ID	Criteria			Number of sequences within sub_database
	Nucleotide sequence length (bp)	Beginning start codons	Ending stop codons	
*intI*1 SDB1	≥1,000	ATG, TTG, GTG	TAA, TAG, TGA	104
*intI*1 SDB2	≥ 900	ATG, TTG, GTG	TAA, TAG, TGA	144
*intI*1 SDB3	≥ 600	N/A	N/A	502
*intI*1_like	≥ 600	N/A	N/A	16
Non-*intI*1 SDB^*a*^	>900	N/A	N/A	1,540

^*a*^ SDB, sub-database.

The *IntI*1 protein database was curated by discarding duplicate protein sequences (*n = 1*) from the 922 *intI*1 protein sequences (Fig. 1). Retained *IntI*1 protein sequences were then compared to the reference *IntI*1 protein sequence of pVS1 plasmid (AAA25857.1) using NCBI BlastP, to ensure *intI*1 nucleotide sequences used for *in silico* assessment of primer and probe sequence coverage were indeed *intI*1 sequences as suggested by Roy et al. (24). Furthermore, protein sequences whose percentage identity to the reference pVS1 *IntI*1 plasmid protein sequence was ≥98%, were characterized as *IntI*1 sequence, while sequences whose protein similarity to the reference pVS1 *IntI*1 plasmid protein sequence were <98% were categorized as *IntI*1-like protein sequences (24). Moreover, the protein sequence identified as *IntI*1-like was manually checked to ensure the percentage similarity score to the pVS1 protein sequence reported by NCBI was not due to missing sequence caused by the alignment of a partial sequence to a complete length sequence. As such, protein sequences (*n = 2;* WP_058137959.1 and WP_058135314.1) incorrectly identified as *IntI*1-like were added to the *IntI*1 protein database (Fig. 1).

Finally, three *intI*1 gene nucleotide sub-databases (SDB1, SDB2, and SDB3) were created for robust primer analysis based on the criterion specified in Table 1.

SDB1 (*n = 104*) contained full-length *intI*1 sequences ≥ 1,000 bp, confirmed by the presence of a start and stop codon; SDB2 (*n = 144*), contained full-length *intI*1 sequences ≥ 900 bp confirmed by the presence of a start codon and stop codon. Sequences within SDB1 are all present in SDB2. The final *intI*1 sub-database (SDB3, *n = 503*) contained both complete and partial sequences (Fig. 1; Table 1). All sequences within SDB1 and SDB2 were also present within SDB3. The *intI*1-like (<98% similarity to pVS1 on protein level) sub-database contained both complete and partial-length sequence (*n = 15*; Fig. 1; Table 1). The non*-intI*1 database contained 1,540 integrase sequences of other classes (Fig. 1).

In parallel, the non-*intI*1 sequence sub-database was constructed from the 2,462 *int*I of other class protein sequences by applying a ≥300 amino acid length thresholds (900 bp nucleotide length) to filter out shorter-length protein sequence (Fig. 1; Table 1). Retained protein IDs for the *intI*1, *intI*1-like, and non-*intI*1 sequences were then used to manually obtain the nucleotide sequences from NCBI in Fasta format.

To summarize, *intI*1 sequences from this study were defined as *intI*1 protein sequences whose percentage identity shared a ≥ 98% similarity to pVS1 *intI*1 plasmid protein sequence (AAA25857.1), while *intI*1-like sequences were defined as *intI*1 protein sequences sharing a < 98% similarity to pVS1 *intI*1 plasmid protein sequence (Fig. 1).

#### Primer evaluation

Published *intI*1 primers were analyzed as primer pair (Table S1), using Primer Prospector (25), to evaluate coverage and specificity against constructed integrase sub-databases (Fig. 1). The analyze_primers.py function with the default settings on Primer Prospector was used to generate an alignment profile file for each primer against unaligned individual nucleotide sequence in each test sub-databases. For each primer alignment to a nucleotide sequence, a weighted score (WS) was given.

#### Overall WS was calculated as

Non-3′ mismatches * 0.4 per mismatch +3′ mismatches * 1.0 per mismatch +Final 3′ base mismatch * 3.0 per mismatch +non-3′ gaps * 1.0 per gap +3′ gap * 3.0 per gap.

The first five bases of the primer and the target sequence were defined as the 3′ end and thus, mismatches within these bases were termed 3′ mismatches. The remaining bases of the primer and the target sequence were defined as the non-3′ end. Therefore, mismatches within these non-3′ end bases were regarded as non-3′ mismatches. Gaps in the alignment of the primer and the target sequence in the first five bases were termed 3′ gaps while gaps in the alignment for the remaining primer and template sequence were known as non-3′ gaps. The lower the WS, the better the compatibility between the primer and target DNA sequence, a 0 score indicates perfect alignment. Primer Prospector will force a primer sequence to bind anywhere within the target sequence even if the primer binding site is unavailable to generate a WS for the primer. As such, the primer binding orientation of each analyzed primer pair was checked for each sequence using R (R Core team 2022). In addition, the seqnir package (26) in R was used to load the DNA/protein sequences. The mean WS for the forward and reverse primer for the primer pairs with the correct binding orientation was noted and a WS plot of each primer set was generated using the “ggplot2” R package (27). A detailed description of the primer evaluation is in supplementary material 2.1.

In the case of primer pairs that incorporated a TaqMan hydrolysis probe, the primer-probe-binding-orientation (forward, probe, and reverse) against each unaligned sequence was first verified, for each unaligned sequence by checking the hit positions of the forward, probe, and reverse primer sequence in R. Unaligned sequences with correct primer-probe orientation were subsequently retained and analyzed in the manner as described in supplementary material 2.1.

#### Design of a new *intI*1 primer set and TaqMan-minor-groove binder (TaqMan-MGB) probe

To improve *intI*1 sequence coverage and specificity for Q-PCR the *intI*1 primer set, F3-R3 (Rosewarne et al., (28), Table S.I) was modified to generate a new *intI*1 primer incorporating an MGB TaqMan probe set (*intI*1 DF-DR, Table S.I) following guidelines for primer-probe design outlined by McKew and Smith (29). An MGB probe of 15 bp was designed using Primer Express software (version 3.0.1; Applied Biosystems). A detailed protocol of the MGB probe design can be found in supplementary material 2.2. Primer and probe sequences were BLAST searched (BLASTN) to validate the sequence specificity. Specificity and coverage of the newly designed primer and probe set were tested as detailed above with Primer Prospector.

### Validation of selected *intI*1 primers from *in silico* analysis on wastewater samples

#### Optimization of selected primer sets for Q-PCR

The amplicon produced from selected primers for laboratory validation was assessed *in silico* first using sequences within SDB1 and then in the laboratory by endpoint PCR. Selected *intI*1 primer sets that resulted in the correct size amplicon were further optimized for Q-PCR assays (Table 2). Q-PCR standard curves were constructed by amplifying a synthetic *intI*1 gene fragment (Integrated DNA Technologies) containing the primer binding site for all selected primers (Fig. S2). The insert fragment was amplified by PCR using T7 forward (5′-TAATACGACTCACTATAGGG-3′) and M13 reverse (5′-CAGGAAACAGCTATGAC-3′) primers. Reaction volume and condition in supplementary material 2.3. Resulting amplicons were purified, and size selected with the Agencourt AMPure XP beads (Beckman Coulter, Brea, CA, USA) per the manufacturer’s recommendation, using a 1:1 ratio of beads volume to PCR product volume, and eluted in a 25 µL volume nuclease-free water. Purified products were quantified fluorometrically using Qubit (Invitrogen, according to the manufacturer’s recommendations), and the gene copy number was determined using EndMemo DNA copy number calculator (http://endmemo.com/bio/dnacopynum.php). The purified concentrated stock was subsequently diluted to 10^9^ copies/μL, followed by a 5-, 10-fold serial dilution (10^7^–10^3^ copies/μL) for amplification by Q-PCR. A standard curve was obtained by plotting the average of each triplicate threshold cycle (Cq) against the log10 of standard concentration (copies/μL). Standard curve descriptors including efficiency, slope, *y*-intercept, and *R^2^* are reported (30).

**TABLE 2:** Primers and probe sets selected and optimized for Q-PCR to quantify *intI*1 gene copies from wastewater^*a*^

Primer ID	Sequence (5′- 3′)	Orientation	Target (length)	Assay type	Q-PCR	Experimental cycle condition	Q-PCR standard curve descriptors					Reference
							Efficiency (%)	*R* ^2^	Slope	Intercept	NTC	
DF DR DF-P	TTCTGGAAGGCGAGCATC TGCCGTGATCGAAATCC Fam-TGACCCGCAGTTGCA-MGB Eclipse	Forward Reverse Probe	*intI*1 (108 bp)	MGB TaqMan probe	10 µL 2× iTaq Universal Probes Supermix (Bio-Rad); 0.8 µL of each primer (10 µM); 0.4 µL probe (10 µM); 1.8 µL MgCl_2_ (final concentration = 3 µM); 4.2 µL nuclease-free water	PCR**:** 95°—15 min; [94°—30 s; 61°—30 s; 72°—30 s] ×35; 72°—10 min Q-PCR: 95°—10 min; [95°—30 s; 60°—60 s, plate read]×45 MiSeq Sequencing 1st step PCR: 95°—15 min; [94°—30 s; 61°—30 s; 72°—30 s]×25; 72°—10 min 2nd-step PCR: 95°—15 min; [95°—30 s, 55°—30 s, 72°—30 s)×8; 72°—5 min	95.7	0.999	3.43	37.71	36.9	This study
F3 R3	TTTCTGGAAGGCGAGCATCGTTTG TGCCGTGATCGAAATCCAGATCCT	Forward Reverse	*intI*1 (109 bp)	SYBR green	10 µL 2× QuantiTect SYBR Green PCR Master Mix (Qiagen); 1 µL of each primer (10 µM); 0.4 µL MgCl_2_ (final concentration = 3 µM); 5.6 µL nuclease-free water	PCR: 95°—15 min; [94°C—30 s; 60°—30 s; 72°—30 s]× 35; 72°—10 min Q-PCR: 95°—15 min; [94°—15 s; 65°—30 s; 72°—30 s, plate read] ×40; melt curve: 65°−95° (0.5° increment/5 s) MiSeq Sequencing 1st step PCR: 95°—15 min; [94°—30 s; 61°—30 s; 72°—30 s] ×25; 72°—10 min 2nd-step PCR: 95°—15 min; [95°—30 s, 55°—30 s, 72°—30 s) ×8; 72°—5 min	91.64	1	−3.54	35.71	36.2	(28)
F7 R7 F7-P	GCCTTGATGTTACCCGAGAG GATCGGTCGAATGCGTGT 6Fam-ATTCCTGGCCGTGGTTCTGGGTTTT-BHQ1	Forward Reverse Probe	*intI*1 (196 bp)	TaqMan probe	10 µL 2× iTaq Universal Probes Supermix (Bio-Rad); 0.8 µL of each primer (10 µM); 0.4 µL probe (10 µM); 1.8 µL MgCl_2_ (final concentration = 3 µM); 4.2 µL nuclease-free water	PCR: 95°—15 min; [94°—30 s; 60°—30 s; 72°—30 s] ×35; 72°—10 min Q-PCR: 95°—10 min; [95°—30 s; 60°—60 s, plate read] × 45 MiSeq Sequencing 1st-step PCR: 95°—15 min; [94°—30 s; 60°—30 s; 72°—30 s] × 25; 72°C—10 min 2nd-step PCR: 95°—15 min; [95°—30 s, 55°—30 s, 72°—30 s) ×8; 72°—5 min	91.29	0.997	3.55	39.6	0	(31)

^*a*^ F = forward primer; R = reverse primer; P = probe; bp = base pair.

### Application of selected *intI*1 primers from SST and CST wastewater samples

#### Solar and conventional tank sampling

Two household solar septic tank (SST; SST01 and SST07) units and three conventional septic tank (CST; two household tanks and one healthcare tank) units, operational within the Pathum Thani province and Samut Prakan province, Thailand, were sampled between April 2018 and September 2019 (Table 3).

**TABLE 3:** Selected sample timepoint for each septic tank investigated^*a*^ ^*,^b^*^

Reactor type	Reactor ID	April 2018	May 2018	June 2018	November 2018	March 2019	June 2019	July 2019	August 2019	September 2019
Solar septic tank (SST)	SST-01	EFF/SLG	EFF/SLG	EFF/SLG						
	SST-07	EFF/SLG			EFF/SLG	EFF/SLG				
Conventional septic tank (CST)	CST-P3						INF/EFF/SLG	INF/EFF/SLG	INF/EFF/SLG	
	CST-J6						EFF/SLG	EFF/SLG	EFF/SLG	
	CST-HC2								EFF/SLG	EFF/SLG

^*a*^ INF = influent; EFF = effluent; SLG = sludge; SST = solar septic tank; CST = conventional septic tank.

^*b*^ Excluded from *intI*1 Q-PCR quantification due to insufficient sample volume but was included in MiSeq amplicon sequencing.

The SST and the household CST units (CST-P3 and CST-J6) have a 1,000 L total working capacity, while the healthcare CST units (CST-HC2; herein referred to as CST-HC) has a 2,000 L total working capacity each. Each tank was buried to approximately 1.5 m below ground level; with the tank surface (lid) at ground level, and so exposed to atmospheric temperatures (7). The sampling approach used is described in supplementary material 2.4 and is outlined elsewhere (7). In total, 100 mL of effluent and 40 mL sludge was sampled from the SST and CSTJ7 and CST-HC, while 100 mL of influent was also collected from CST-P3. 40 mL of sludge was sampled from each reactor. All samples were pelleted for DNA extraction. For the sludge, DNA was exacted from 0.5 g pellet. The months for sampling the SST were selected based on the highest recorded internal temperature of the 12-month sampling campaign conducted.

#### DNA extraction

From each sample, DNA extraction was performed with the DNeasy PowerSoil Kit (Qiagen), in accordance with the manufacturer’s instructions. Integrity of extracted genomic DNA was assessed *via* agarose gel electrophoresis and DNA concentration was quantified fluorometrically using the Qubit (Invitrogen) according to the manufacturer’s instructions.

#### Q-PCR quantification of *intI*1 gene from wastewater

*intI*1 genes were quantified from septic tank wastewater samples from Thailand (Table 3) using optimized Q-PCR conditions for the three selected *intI*1 primer pairs (DF-DR, F3-R3, and F7-R7). For each primer set, Q-PCR amplification was carried out in a 20 µL volume reaction using 2 µL (1:50 diluted) template DNA. Reaction volume, conditions, primer sequences, and probe type for the three selected optimal *intI*1 primer pairs are detailed in Table 2. Triplicate/duplicate no template control (NTC) was included for each primer set. Reactions were performed on the Bio-Rad CFX96 Touch Real-Time PCR Detection System and analyzed with the Bio-Rad CFX Manager 3.1 software. Melt curve analysis was performed, for the SYBR Green assay, from 65°C to 95°C with 0.5°C increments every 5 s, and a single peak was confirmed to ensure assay specificity.

Statistical analyses were performed in R (R Development Core Team, 2008). Two-way analysis of variance (ANOVA) followed by a Turkey HSD post hoc test was employed to compare *intI*1 gene abundance for each of the sample types (influent, sludge, and effluent) and reactor type (CST and SST) for each primer set. Finally, a Pearson correlation analysis was applied to calculate the relationship between the abundance of *intI*1 detected between each primer set.

#### MiSeq amplicon sequencing

The specificity of the selected *intI*1 primer sets (DF-DR, F3-R3, and F7-R7) used to quantify the *intI*1 gene from septic tank sludge and wastewater was confirmed by Illumina MiSeq amplicon sequencing of the *intI*1 gene from 31 wastewater samples (Table 3) using the optimized endpoint PCR conditions outlined in Table 2. A two-step PCR was performed to barcode samples as detailed previously (32, 33). A detailed description of the method is provided in supplementary material 2.5.

#### Bioinformatics

Primer sequences were used to extract the *intI*1 gene from the resulting reads, particularly for shorter primer pairs, using the Cutadapt algorithm (34). Abundance tables were then generated by constructing amplicon sequencing variants (ASVs) using the Qiime2 pipeline and the DADA2 algorithm (35) with details given at https://github.com/umerijaz/tutorials/blob/master/qiime2_tutorial.md. Constructed ASVs were blast searched on NCBI, and the closest hit sequences were retrieved for each ASV. The phylogenetic distance between sequences was investigated. First, a multiple sequence alignment of ASV sequences retrieved NCBI sequences, complete length *intI*1 and *int*I1-like and an *intI*3 (class three integrase gene; nucleotide ID: AY219651.1) sequence was done using MAFFT (36) for each primer set. Aligned sequences were visualized in BioEdit (version 7.0.5.3) (23) and trimmed to retain only aligned regions without gaps. Phylogenetic trees were constructed using a maximum likelihood approach with a generalized timer-reversible substitution model implemented in RAxML version 8 (37). Consensus trees were calculated after 1,000 bootstrapping permutations.

The phylogenetic tree of the trimmed and aligned sequence, for each primer pair, was constructed with RAxML (38). A heat tree of the constructed ASVs, after log2 transformation of ASV abundance per sample for each primer set, was mapped to analyzed samples, colored, and visualized using the ggtree package (39). The tip of the tree was colored based on the sequence isolation source.

### Validation of selected primers to quantify *intI*1 mRNA transcripts from environmental samples

#### Sample collection, filtration, and DNA/RNA co-extraction

As the septic tank wastewater samples were previously collected and only DNA extracted and stored at −80°C, they were not suitable for RNA analysis (33). Therefore, we tested the suitability of the optimized primer sets to detect *intI*1 mRNA using freshly collected environmental samples of river water collected from the Kevin River, Glasgow (UK), to determine whether *intI*1 mRNA transcripts could be quantified in receiving water bodies. In total, 3 L of surface water was collected in March and April 2022 and filtered through a sterile glass microfiber filter (FisherBrand MF200; retention 1.2 µm) and onto a 0.22-µm Sterivex filter. Filters were immediately extracted from or frozen at −80°C for later use.

DNA-RNA co-extraction was carried out according to the protocol previously described (33, 40, 41), with a minor modification to the bead-beating time (45 s) as outlined by Lim et al. (42). Detailed description of this method is outlined in supplementary material 2.6. Briefly, RNA was prepared from the DNA-RNA co-extract by DNase treating with Turbo DNase Kit (Ambion) in accordance with the manufacturer’s recommendation, with modification to the incubation time and volume of DNase added as previously described (33), 1 µL DNase volume was added and incubated at 37°C for 1 hour, followed by further addition of 1 µL DNase and a re-incubation at 37°C for another hour. Detailed protocol is found in supplementary material 2.7.

#### RT-Q-PCR quantification of *inti*1 genes and transcripts from river water

Q-PCR DNA standard curve was constructed as above (see above section Optimization of selected primer sets for Q-PCR). For each primer set*, intI*1 cDNA and DNA Q-PCR amplification was carried out in a 20 µL volume reaction using 2 µL (1:2 and/ 1:5 diluted) template DNA/cDNA. In addition, two priming strategies, gene-specific (GS) and/or random (RH) priming, were used to reverse transcribe *intI*1 mRNA to cDNA. Detail of this approach is provided in supplementary material 2.7. Q-PCR volume, conditions, primer sequences, and probe type for the three selected optimal *intI*1 primer pairs are the same as specified in Table 2. Reactions were performed on the Bio-Rad CFX96 Touch Real-Time PCR Detection System and analyzed with the Bio-Rad CFX Manager 3.1 software. Melt curve analysis was performed, for the SYBR Green assay, from 65°C to 95°C with 0.5°C increments every 5 s, and a single peak was confirmed to ensure assay specificity. Standard curve descriptors including efficiency, slope, *y*-intercept, and *R^2^* are reported (Table 4).

**TABLE 4:** *intI*1 mRNA transcripts copies/ng DNA^*a*^

Primer set	Assay	Priming strategy	Mean gene copy number (*n = 3*)				Q-PCR standard curve descriptors				
			1 in 2 dilution		1 in 5 dilution						
			cDNA	DNA	cDNA	DNA	Efficiency (%)	*R* ^2^	Slope	Intercept	NTC
F3-R3	SYBR Green	GS	1.24 × 10^4^ ± 7.83 × 10^2^	1.63 × 10^4^ ± 9.15 × 10^3^	N.T	N.T	92.4	1	3.52	35.84	0
DF-DR	SYBR Green	GS	2.62 × 10^3^ ± 9.63 × 10^2^	8.99 × 10^4^ ± 3.49 × 10^4^	4.17 × 10^3^ ± 1.2 × 10^3^	1.12 × 10^5^ ± 4.6x10^3^	94.3	0.997	3.47	35.25	0
		RH	N.T		2.01 × 10^3^ ± 5.8 × 10^2^						
	TaqMan	GS	N.T	N.T	4.33 × 10^3^ ± 2.98 × 10^2^	1.83 × 10^5^ ± 2.72 × 10^4^	94.9	0.999	3.45	37.49	0
		RH	N.T		3.61 × 10^3^ ± 2.67 × 10^2^						
F7-R7	SYBR Green	GS	5.43 × 10^3^ ± 5.12 × 10^2^	1.23 × 10^5^ ± 7.79 × 10^3^	8.41 × 10^3^ ± 4.35 × 10^2^	1.54 × 10^5^ ± 5.25 × 10^3^	90	0.999	3.59	35.35	41.31
		RH	N.T		1.44 × 10^3^ ± 2.89 × 10^2^						
	TaqMan	GS	N.T	N.T	ND	1.63 × 10^5^ ± 3.66 × 10^3^	93	0.999	3.5	37.41	37.64
		RH	N.T	N.T	ND						

^*a*^ GS = gene-specific priming; RH = random hexamer priming; ND = not detected; N.T = not tested.

## RESULTS

### *intI*1 primer evaluation

#### Evaluation of primers for coverage

In total, 64 different *intI*1 primer sets, including 4 TaqMan primer-probe sets, were retrieved from the systematic review (Table S1). In addition, the primer and probe set designed in this study were included in the analysis, resulting in 65 primers evaluated (Table S1). Primers were initially aligned against the reference *P. aeruginosa* plasmid pVS1 nucleotide sequence (M73819.1) (Fig. S1) and renamed for ease of identification Table S1). Next, primers were aligned against the SDB1 (full-length *intI*1 database) to ensure binding sites were present (in forward or reserve orientation) and that the expected amplicon size would be generated. From this, 10 primer sets were discarded, which included two sets (F61-R61 and F64-R64) that were not *intI*1 primers (Table S2). The F61-R61 primer set targeted the *aadA1a* aminoglycoside adenylyl transferases gene (43, 44), while the F64-R64 primer pair targeted the class two integron-integrase gene (*intI*2) (45). In addition, these primer sets (F61-R61 and F64-R64) aligned poorly to the reference *P. aeruginosa* pVS1 *intI*1 nucleotide sequence (data not shown) and had no hit (High WS) with complete length *intI*1 sequences within SDB1 (Table S2).

Of the remaining 55 primer pairs, when aligned to SDB1 (complete length sub-database *n = 104*) ≥97%–100% of sequences had the correct primer binding orientation (Table S3). In addition, the majority of these primers (49 sets; 89%) amplified 69%–100% of sequences in the correct primer binding orientation with 0 mismatch (Fig. S3A and B; Table S3). One primer (F40-R40) performed poorly, amplifying only 0.9% (*n = 1* amplicon) sequences with the correct primer binding orientation at 0 mismatch (Fig. S3; Table S3) and was removed from further consideration.

Primer coverage was then tested against the other *intI*1 complete length and partial length sub-databases, SDB2 (*n = 144*) (Fig. S3 C and D) and SDB3 (*n = 503*) (Fig. S4), respectively. Here, the number of sequences with the correct primer binding orientation declined (SDB2: 79%–100%; SDB3: 41%–100% of the sequences had the correct primer binding orientation), as did the number of amplicons amplified with a 0 mismatch (SDB2: 50%–99%; SDB3: 16%–99%) for the correct primer binding orientation sequences (Fig. S3 and S4; Table S3).

Five (9%) primer sets produced no amplicon at a WS of 0 across the three *intI*1 sub-databases (Fig. S3 and S4; Tables S3) and were removed from further consideration. This included one set (F29-R29) which performed optimally when allowing a single mismatch at the 5′ end (WS = 0.4) (Table S4), but as there were several primers with better coverage at a WS of 0, this primer set was removed. In summary, a further six primer sets (F9-R9, F29-R29, F40-R40, F53-R53, F56-R56, and F62-R62) were discarded from the primer coverage analysis.

Of the 49 amplicon-producing primers at 0 WS retained, 10 primer sets (DF-DR, F1-R1, F3-R3, F7-R7, F13-R13, F16-R16, F31-R31, F35-R35, F57-R57, and F60-R60) consistently had a high number (99%–100%) of sequences with the correct primer-binding orientation and amplified ≥97% amplicons within the complete length sub-databases (SDB1: *n = 104* and SDB2 *n = 144*). Moreover, these primers consistently had a low mean WS for the forward and reverse primer within each pair (Table S3). As such, these 10 primers were considered the best-performing *intI*1 primer sets.

Five primer sets analyzed in this study incorporated a TaqMan probe (Table S5), two of which (DF-DR and F7-R7 sets) were among the best-performing primer sets. Of these, the DF-P-DR primer-probe set, designed in this study, consistently produced the highest number of amplicons at 0 WS across the three *intI*1 sub-databases with 102 (98%), 142 (99%), and 494 (99%) of sequences amplified within SDB1, SDB2, and SDB3, respectively (Table S5). In addition, allowing for a single non-3′ mismatch (WS = 0.4) between primer and probe, resulted in all sequences with the correct primer-binding orientation to be amplified across the three *intI*1 sub-databases (Table S5).

Conversely, primer-probe set F46-P-R46 performed the worst, of the five primer-probe combinations assessed, with only 34 (33%), 41 (32%), and 88 (23%) of sequences with the correct primer-binding-orientation amplified at 0 WS within SDB1, SDB2, and SDB3, respectively. Nonetheless, allowing for an increased WS of 1 (i.e., mismatch caused by either a single 3′ end mismatch, a non-3′ end gap or two non-3′ mismatches) resulted in a significant increase in the number of amplicons amplified across *intI*1 sub-databases [SDB1: *n = 95* (93%), SDB2: *n = 121* (93%), and SDB3: 360(95%)] (Table S5).

The F7-P-R7 primer-probe set (commonly used TaqMan assay in *intI*1 gene study), F10-P-R10, and F38-P-R38 primer-probe sets showed similar coverage to each other, but lower than DF-P-DR set, with 92 (88%), 91 (88%), and 92 (88%) of amplicons amplified at 0 WS within SDB1 respectively (Table S5). However, the F7-P-R7 primer-probe set amplified the highest number of amplicons at 0 WS (or second highest after the DF-DR set) for the correct primer binding sequences across the other two *intI*1 sub-databases [SDB2: *n = 131* (91%), SDB3: *n = 454*(96%)] among the three primer sets (Table S.5). As such, the DF-P-DR and F7-P-R7 primer-probe sets were put forward as the top performing primer-probe set.

#### Evaluation of primers for specificity

The primer sets were tested for specificity against the *intI*1-like (*n = 15*) and non-*intI*1 (*n = 1540*) sub-databases, respectively (Fig. S5; Table S3). Here, the aim was for the primers to amplify the least amount of non-target sequence reflected by a higher forward and reverse primer WS for sequences where primers bind in the correct orientation. The 10 best-performing primer sets identified above were focused on.

For the best-performing primer sets, the number of sequences with correct primer-binding orientation ranged from 67% to 100% and 41% to 65% for the *intI*1-like and non-*intI*1 sub-databases, respectively, with 57%–80% and 0% of these correct primer-binding orientation sequences amplified at 0 mismatch in *intI*1-like and non-*intI*1 sub-databases, respectively (Fig. S5, Table S3).

Of these best-performing sets, the F16-R16 primer set amplified the highest number of *intI*1-like amplicons (*n = 11*, 79%) at 0 mismatch and was removed, while the primer pair F57-R57 amplified the lowest number of *intI*1-like sequence (*n = 7*) (Fig. S5A, Table S3). The incorporation of a TaqMan probe generally improved primer specificity; however, two of the primer sets that incorporated a TaqMan probe (DF-P-DR and F7-P-R7) both amplified *intI*1-like sequences. The number of *intI*1-like amplicons amplified by the DF-P-DR (*n = 9*) and F7-P-R7 (*n = 8*) primer-probe sets at a 0 WS were similar. Of note, while the 10 best-performing was focused on, the other primer sets analyzed (see section Evaluation of primers for coverage) also amplified *intI*1-like sequences (Fig. S5A; Table S3).

Next, the nine remaining primer sets were tested against the non-*intI*1 sequences (Fig. S5B and C). None of the primers amplified the non-*intI*1 sequence at a 0 mismatch. In general, primers only produced amplicons from the non-*intI*1 database with very high weighted scores (sum of forward and reverse primer mean WS ranged: 8.39–11.6) (Fig. S5B and C; Table S3). However, the primer pairs F1-R1 (WS: 2) and F13-R13 (WS: 3.2) performed worst, having the lowest WS required to amplify at least one non-*intI*1 target. As such, were removed from further analysis, leaving seven sets (DF-DR, F3-R3, F7-R7, F31-R31, F35-R35, F57-R57, and F60-R60) to be considered the best overall performing *intI*1 primer sets in terms of coverage and specificity.

#### Recommendation of optimal primer sets for *in situ* laboratory validation and *in silico* amplicon size distribution

From the initial 65 primer sets, seven (DF-DR, F3-R3, F7-R7, F31-R31, F35-R35, F57-R57, and F60-R60) were identified that had high coverage in our *intI*1 database, but low specificity to the non-*intI*1 database, indicating they are good primer sets targeting a broad range of *intI*1 targets while discriminating against non-*intI*1 sequences. Two published sets (F3-R3 and F7-R7) were selected (Table S3), as they not only had the highest WS required to amplify non-*intI*1 target [i.e., needed the highest number of mismatches to target the sequence; F3-R3- WS: 5.2 (2 non-*intI*1 amplicons), F7-R7- WS 5.2: (six non-*intI*1 amplicons)] but also had short amplicons (100–200 bp), making them ideal for both Q-PCR and high-throughput amplicon sequencing. In addition, each of these selected primer sets targeted a different region of the *intI*1 gene and was commonly used within the literature (28, 31, 46 – 49). F7-R7 incorporated a TaqMan probe. The primer and probe set, DF-P-DR, designed in this study, were also included resulting in three *intI*1 primer sets selected for laboratory validation.

### Application of selected *intI*1 primers on septic wastewater samples

#### Q-PCR quantification of *intI*1 gene from Thai-Septic Tanks wastewater

The three selected and optimized *intI*1 primer sets and probes (DF-DR, F3-R3, and F7-R7; Table 2) were used to quantify *intI*1 gene abundance across 30 septic tank wastewater samples (influent, sludge, and effluent) from CST-household, CST-healthcare, and SST-household reactors (Table 3).

Each of the standard curves from all three primer sets had high efficiencies which ranged from 91.29% to 95.7%, *y*-intercepts of 35.71 to 39.6, the slope of −3.43 to −3.55 and a No Template Control Ct from undetected to 36.9 (Table 2).

**Fig 2:**
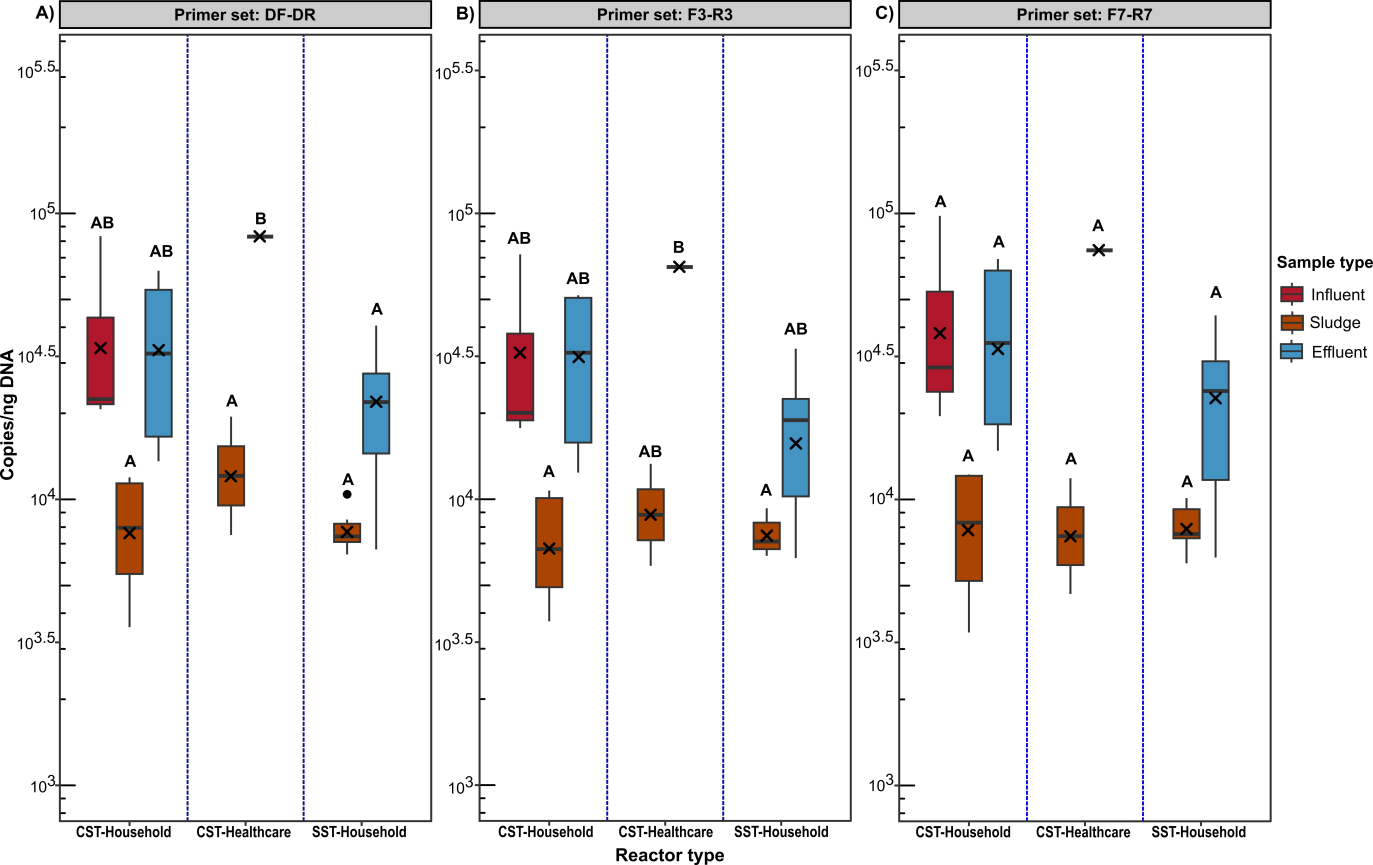
Impact of primer choice on the quantification of *intI*1 gene copies from CST-household, CST-healthcare, and SST-household septic tank wastewater reactors, and three wastewater sample types (influent, effluent, and sludge). Results of the two-way ANOVA analysis showed a statistically significant difference in *intI*1 gene copies quantified between reactor types and sample types. For each primer set, a boxplot sharing the same letter indicates no statistically significant difference at *P*-value > 0.05, while the boxplot with different letters indicates a statistically significant difference at *P*-value < 0.05. A statistically significant difference in *intI*1 gene abundance between primer sets for the same sample was not observed (*P*-value > 0.05; see supplementary table s.vi). X icon indicates mean *intI*1 copy number/ng DNA. The black dot indicates the data outlier.

Of note, between primer sets, there was no difference in *intI*1 gene abundance for the same sample type (influent, sludge, and effluent) (*P*-value > 0.05, Fig. 2; Table S6), and Pearson correlation coefficient analysis indicated that the *intI*1 gene copy number amplified by each of the primers was highly correlated [*r* = 0.982 (*P*-value < 0.001), *r* = 0.99 (*P*-value < 0.001), and *r* = 0.993 (*P*-value < 0.001) for DF-DR and F7-R7, DF-DR and F3-R3, and F3-R3 and F7-R7 primer sets, respectively]. As such, each primer set resulted in the same overall pattern of *intI*1 gene abundance, with higher *intI*1 gene copies/ng DNA observed in the influent (DF-DR: 4.22 × 10^4^ ± SD3.57 × 10^4^; F3-R3: 3.66 × 10^4^ ± SD3.06 × 10^4^; F7-R7: 4.89 × 10^4^ ± SD4.29 × 10^4^ copies/ng DNA) > effluent (DF-DR: 3.33 × 10^4^ ± SD2.30 × 10^4^; F3-R3: 2.88 × 10^4^ ± SD1.93 × 10^4^; F7-R7: 3.53 × 10^4^ ± SD2.39 × 10^4^ copies/ng DNA) > sludge (DF-DR: 8.81 × 10^3^ ± SD3.94 × 10^3^; F3-R3: 7.72 × 10^3^ ± SD 2.65 × 10^3^; F7-R7: 8.24 × 10^3^ ± SD 3.07 × 10^3^ copies/ng DNA) (Fig. 2).

Although a similar overall pattern of *intI*1 gene abundance was observed, the F7-R7 primer set was the only primer set that reported no statistical difference (*P*-value > 0.05) in gene abundance between samples (influent, sludge, and effluent) and reactors (CST-household, CST-healthcare, and SST-household), while only DF-DR primer set showed significantly higher *intI*1 gene abundances in the effluent of the CST healthcare than all other samples (Fig. 2).

SST-household units incorporated an internal pasteurization effect and were therefore expected to have lower *intI*1 gene abundance in the effluent. *intI*1 gene abundance per ng of DNA was lower in effluent than in both the CST-household and CST-healthcare tanks for all three primer sets (Fig. 2). However, these differences were only statistically significant for DF-DR primer se (*P*-value = 0.005) between SST-household and CST-healthcare effluent (Fig. 2). Nonetheless, the lower *intI*1 gene abundance quantified in the solar septic tank (SST-household) effluent, albeit only statistically lower in the CST-healthcare for the DF-DR primer set.

Although *intI*1 gene copies in the sludge of the reactors were lower than the effluent, these *intI*1 gene copies were still high, albeit that the gene abundance between the three reactors was marginally different but not statistically significant (*P*-value > 0.05), regardless of the primer set (Fig. 2). However, depending on the primer set used, the reactor with the higher abundance and thus likely to contribute most to the environment changed.

The DF-DR primer set reported the CST-healthcare (1.35 × 10^4^ ± SD8.45 × 10^3^ copies/ng DNA) to be the higher contributor of *intI*1 gene to the environment *via* sludge and the SST-household unit (7.86 × 103 ± SD1.43 × 10^3^ copies/ng DNA) to be the lowest of the three reactors (Fig. 2A), while primer set F3-R3 also indicated the CST-healthcare sludge as the higher contributor (9.58 × 10^3^ ± SD5.27 × 10^3^ copies/ng DNA) of CL1-intgeron to the environment *via* sludge, but reported the CST-household (7.30 × 103 ± SD3.11 × 10^3^ copies/ng DNA) as the least contributor (Fig. 2B). Finally, the F7-R7 primer set revealed the CST-household unit sludge (8.42 × 10^3^ ± SD4.10 × 10^3^ copies/ng DNA) to be the greater contributor of *intI*1 to the environment and the SST-household (8.06 × 10^3^ ± SD1.59 × 10^3^ copies/ng DNA) as the least contributor (Fig. 2C). As such, the SST-household in general had the lowest *intI*1 gene abundances in sludge when primer sets DF-DR and F7-R7 was used, but not when the F7-R7 primer set is used (Fig. 2).

Influent samples were only accessible from the CST units with *intI*1 gene abundance higher in the influent (DF-DR: 4.22 × 10^4^ ± SD3.57 × 10^4^; F3-R3: 3.66 × 10^4^ ± SD3.06 × 10^4^; F7-R7: 4.89 × 10^4^ ± SD4.29 × 10^4^; copies/ng DNA) than effluent (DF-DR: 2.43 × 10^4^ ± SD1.76 × 10^4^; F3-R3: 2.52 × 10^4^ ± SD2.06 × 10^4^; F7-R7: 2.95 × 10^4^ ± SD2.34 × 10^4^ copies/ng DNA), indicating a removal efficiency [mean influent − mean effluent/ mean influent)*100] of 42.42%, 31.15%, 39.67%, for the DF-DR, F3-R3, and F7-R7 primer sets, respectively (Fig. 2).

In summary, primer sets used did not change the overall pattern of *intI*1 gene abundances nor did it result in statistical difference (*P*-value > 0.05) in *intI*1 gene abundance for the same sample type (influent, sludge, and effluent) quantified with the different primers. However, comparing samples within the same primer set did sometimes result in statistical differences between samples., which could alter the interpretation of the risk of *intI*1 gene abundances, and, in turn, AMR pollution to the environment.

#### MiSeq amplicon sequencing

MiSeq amplicon sequencing was undertaken on all septic tank samples (*n = 31*; Table 3), to confirm the specificity of the selected *intI*1 primers (Table 2) and assess the diversity of the *intI*1 amplicons retrieved from the septic tanks. Overall, the number of unique ASVs generated by each primer set was low, with 3 ASVs for DF-DR; 4 ASVs for F3-R3; and 11 ASVs for the F7-R7 primer set (Table S7).

Overall, the number of unique ASVs generated by each primer set was low, with 4 ASVs for DF-DR; 4 ASVs for F3-R3; and 11 ASVs for F7-R7 primer set. One ASV for the DF-DR primer set was removed due to cross-contamination with the F3-R3 set, owing to the high similarity of both primer sets and a similar target region. This excluded ASVs from the DF-DR primer set that had a maximum abundance of 8 ASVs across three samples (a total of 20 ASVs removed). In summary, 3, 4, and 11 ASVs were generated for DF-DR, F3-R3, and F7-R7 primer sets, respectively. The summary statistics of the sequencing output is provided in Table S7.

**Fig 3:**
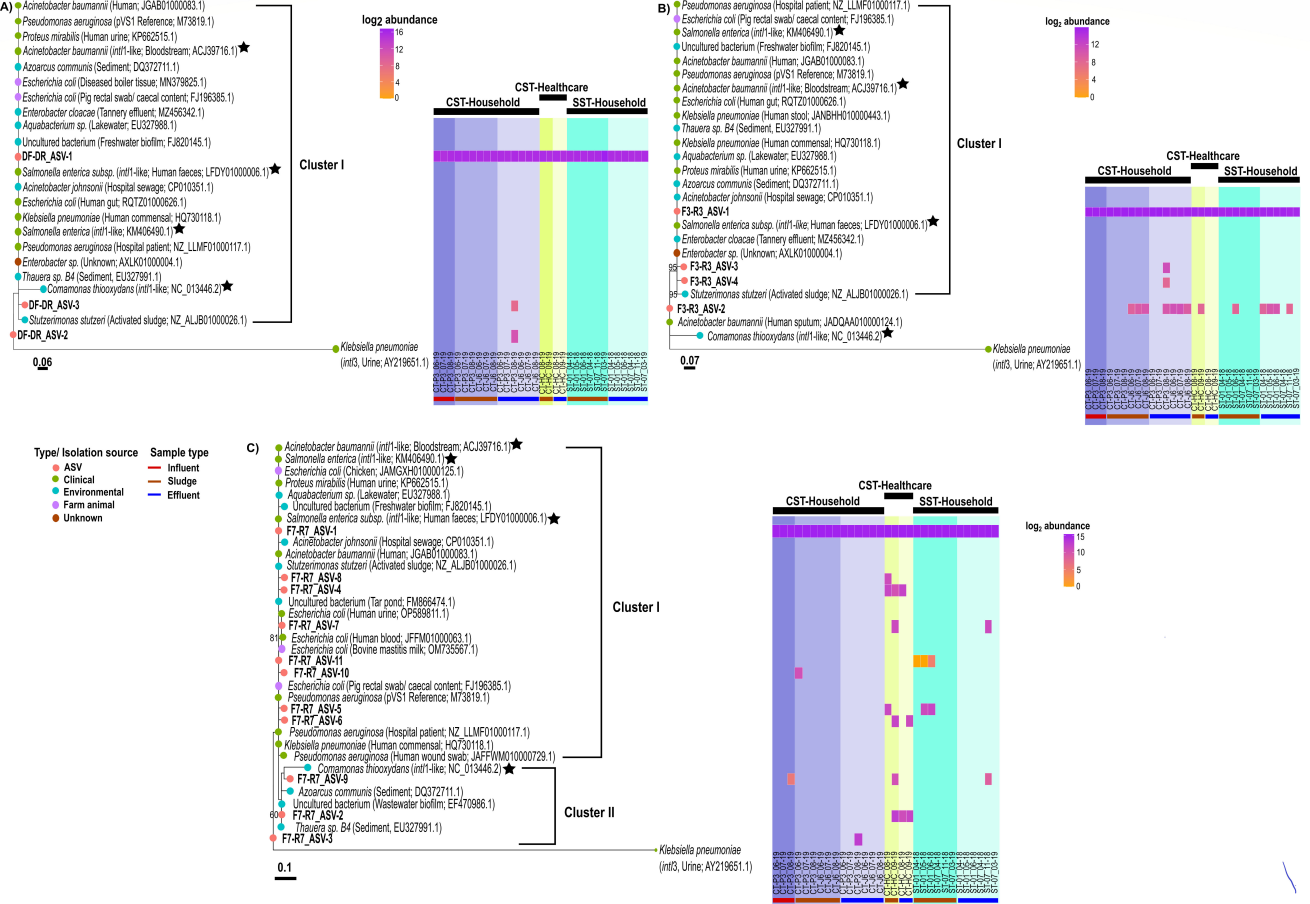
Detected ASVs abundance in Thai septic tank wastewaters (SST-household, CST-healthcare, and SST-household) by the DF-DR (**A**), F3-R3 (**B**), and F7-R7 (**C**) *intI*1 primer sets. Generated ASVs coupled with known and unknown *intI*1 within SDB1 (*n = 104*), best hit NCBI sequences, and *intI*1-like sequences were aligned with Mafft, trimmed to only aligned region with no gaps, and phylogenetic tree constructed using the RAxML with 1,000 bootstrap permutations. The number at a node represents a bootstrap value > 50% (from 1,000 permutations). The bootstrap value at node < 50 is not shown. The class 3 integron-integrase (*intI*3) gene (nucleotide ID: AY219651.1), which on the protein level, shared a 60.74% similarity to the pVS1 protein sequence (AAA25857.1) was used as the outgroup. The color of tree tips indicates the isolated source of sequence/ ASVs generated by the primer set. Heatmap shows log2 fold abundance (mean number of ASVs-DF-DR:5.1955 × 10^4^, F3-R3: 4.6602 × 10^4^ and F7-R7: 3.6684 × 10^4^; Table S7) of detected ASVs within each wastewater sample. CTP3 and CTJ6 samples originated from two independent CST-household reactors. CT-HC sample was from a CST-healthcare tank. ST01 and ST07 are two independent SST-household units. The sampling month and year are indicated by the format month_year (i.e., 06_19 = June 2019). CST, conventional septic tank; SST, solar septic tank.

A phylogenetic tree was constructed with the highly conserved *intI*1 sequences from a range of environmental and clinical samples. In addition, *intI*1-like sequences (*n = 4*) were added to the tree to determine whether the primer sets could distinguish between these and *intI*1 sequences (Fig. 3). All *intI*1 and *intI*1-like sequences, including the ASVs found here, showed high sequence similarity to each other, likely owing to the short amplicon region designed over conserved regions (Fig. 3). *intI*1-like sequences clustered among the *intI*1 and ASVs for all three primer sets, indicating that the primer sets could not differentiate between both variants.

All three primer sets amplified an abundant ASV-1 phylotype as the dominant *intI*1 sequence present in all septic tank types and sample types. This ASV was highly similar to *intI*1 and *intI*1-like sequences found in a range of environmental samples including freshwater biofilm, tannery effluent, hospital sewage, and activated sludge (Fig. 3).

For primer set DF-DR, a single cluster of *intI*1 sequences was present, albeit not supported by a bootstrap value, which also contained a second ASV (ASV-3) only present in the CST-household effluent (CST-P3_08–19). A third ASV (ASV-2), again only detected in the CST-household effluent (CST-P3_08–19), clustered outside the main group, highly similar to the *intI*1 sequence from activated sludge, although with a low bootstrap value (Fig. 3A).

For primer set F3-R3, a single cluster was observed, supported by a 95% bootstrap value containing ASV-1, 3, and 4. These clustered with unknown and known *intI*1 from Tannery effluent and activated sludge as well as known *intI*1-like sequences of clinical origin (Fig. 3). While ASV-1 was present in all samples, ASV-3 and 4 were only detected in the CST-household effluent (CST-P3_08–19). Outside of this cluster was ASV-2, highly similar to *intI*1 from *Acinetobacter baumannii*, a clinical pathogenic bacterium. ASV-2 was present in both the CST-household and SST-household tanks sludge and effluent but only present in one CST-healthcare sludge sample (CT-HC_09–19) (Fig. 3B). As primer sets DF-DR and F3-R3 targeted the same region of the *intI*1 (Fig. S1), the ASVs generated by each primer set (DF-DR and F3-R3- ASV-1; DF-DR-ASV-2 and F3-R3-ASV-3; DF-DR-ASV-3 and F3-R3-ASV-4) had 100% sequence similarity to each other but only ASV-1 from each primer set showed a 100% sequence similarity when aligned against the full-length *intI*1 nucleotide sequences (pVS1, M73819.1). In addition, F3-R3-ASV-2 did not align with the full-length *intI*1 with a 100% similarity.

Primer set F7-R7 detected 11 ASVs separated into two clusters. Within cluster I, ASV-1 was present in all samples, clustered with ASV-8, 4, 7, 11, 10, 5, and 6 and was detected in CST-household (sludge), CST-healthcare (sludge and effluent), and SST-household (sludge and effluent) reactors. It clustered with known *intI*1 from sources such as hospital sewage and Tar-Pond but also *intI*1-like sequences. Clustering was not supported by a high bootstrap value. Within cluster II, ASV-9 and 2 were detected in CST-household influent, CST-healthcare sludge and effluent, and SST-household effluent samples and clustered with unknown and known *intI*1 sequence, as well as *intI*1-like sequence, found in environmental source such as sediment and wastewater biofilm. However, clustering was not supported by a high bootstrap value (<50%) (Fig. 3). A final ASV (ASV-3), again only detected in the CST-household effluent (CST-P3_08–19), clustered outside the main group, but was not supported by a high bootstrap value (<50%) (Fig. 3C).

In summary, *intI*1 diversity showed all samples to be dominated by a single ASV-1. It was highly similar to *intI*1 from clinical and environmental samples; however, *intI*1-like samples also clustered with it. CST-household had the highest richness with primer set DF-DR and F3-R3, but a different picture arose with the F7-R7 primer set with the CST-healthcare effluent having the highest *intI*1 diversity.

#### Laboratory validation of selected *intI*1 primers to quantification *intI*1 mRNA transcript from environmental samples

**Fig 4:**
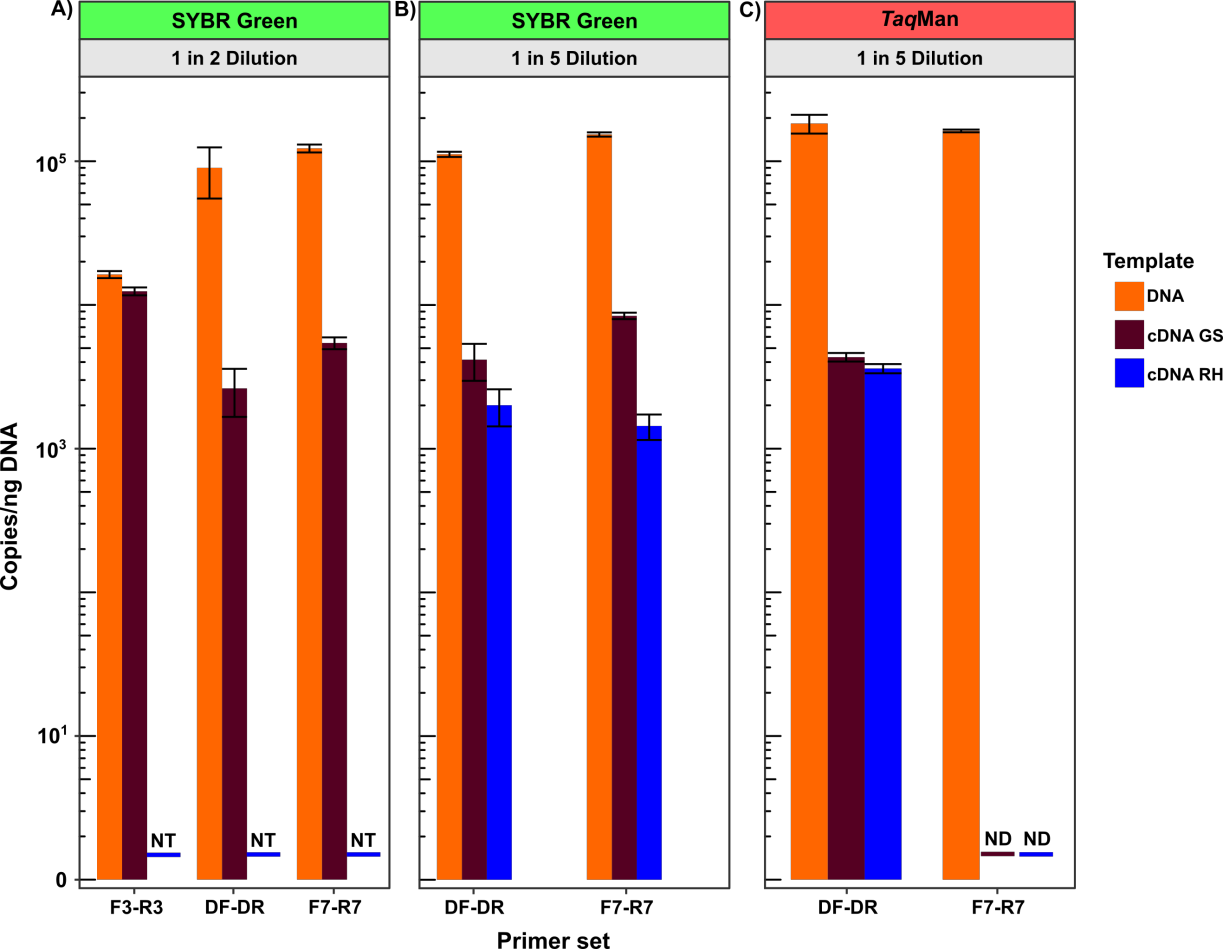
*IntI*1 DNA and mRNA transcript quantified from a river water sample by the three selected *intI*1 primer sets (DF-DR, **F3-R3, F7-R7**).The reverse transcriptase reaction for each primer set was performed with random hexamers (RH) and gene-specific (GS) primers. In addition, TaqMan assays were carried out with (**C**) and without the probes (i.e., SYBR Green) (**A, B**). NT denotes not-tested, and ND denotes non-detected.

As the detection of *intI*1 DNA does not infer integrase activity, each of the validated primer sets was tested for their ability to quantify *intI*1 mRNA transcripts. For this, fresh river water samples were used as the quality of the RNA extracted wastewater nucleic acids may be of poor quality due to long-term storage (33), although the quality was not measured. For each primer set, the reverse transcriptase reaction was carried out with random hexamers (RH) and gene-specific (GS) primers as previous work showed increased specificity with gene-specific priming (50). In addition*,* Q-PCR quantifications were carried out with (Fig. 4C) and without the probes (i.e., SYBR Green) (Fig. 4A and B; Table 4). All primer sets successfully quantified *intI*1 DNA and mRNA from river water, with *intI*1 gene abundances greater than *intI*1 transcripts (Fig. 4).

As previously shown (50), gene-specific priming was more efficient than random hexamer priming. The F7-R7 primer set did not work as a TaqMan probe assay but worked in a SYBR green assay. It should be noted that, while higher *intI*1 transcript copies per ng DNA were quantified by the F3-R3 SYBR green assay (1 in two dilution), direct comparison to DF-DR and F7-R7 cannot be made as they were not done on the same sample. The aim here was simply to demonstrate that the primer sets were able to quantify *intI*1 mRNA transcripts. In summary, the primer sets tested are appropriate to quantify *intI*1 mRNA transcripts from environmental samples, using both gene-specific and random hexamer priming, albeit the TaqMan probe chemistry must be swapped to SYBR green chemistry if the F7-R7 primer set is to be used.

## DISCUSSION

Accurate quantitative data are key to inform evidence-based management strategies and policies to reduce the global AMR burden. Quantitative approach, alongside unified methodologies to enable comparison among data sets, is a powerful tool to enable this. The clinical class 1 integron (CL1-integron) integrase gene (*intI*1) has been proposed as a proxy for inferring potential AMR (8). The first step to investigating the potential for this is to select appropriate primers; however, our systematic literature review revealed over 65 *intI*1 primer sets with little consensus on the best primer to use. Through *in-silico* testing of the published primer sets, in addition to the design of an optimized primer set in this study, we selected three *intI*1 primer sets for laboratory validation and further testing of their specificity on septic tanks from Thailand associated with healthcare and household usage to investigate their contribution in disseminating CL1-intgerons to the environment. This included a novel solar septic tank designed with internal heating ranging from 39°C to 63.6°C in the disinfection chamber. From the 65 primers in the literature, three were selected- two published primer sets, F3-R3 (28) and F7-R7 (31) which have been extensively applied to survey CL1-integron abundance in a range of ecological settings including WWT (48, 49) and agricultural settings (46, 47) and a newly designed primer, DF-DR, modified from the F3-R3 primer set and an MGB probe added to increase specificity showed good coverage and specificity. All were successful PCR and RT-Q-PCR assays.

To confirm their specificity, MiSeq amplicon sequencing of the short amplicons was undertaken from the Thai septic tank samples. While the diversity of the amplicons was low, likely reflecting the short amplicon length, the *intI*1 gene was ubiquitous in our samples supporting previous findings where it was dominant in polluted environments such as WWT (8, 51). The ASVs generated from each primer set were highly similar to *intI*1 and *intI*1-like sequences obtained from known and unknown bacteria, which were isolated from a range of clinical settings (e.g., human commensal) and environmental sources (e.g., wastewater-activated sludge) (Fig. 3). Interestingly, a few of the *intI*1-like sequence characterized were from known bacterial species isolated within clinical context including human feces and bloodstream (Fig. 3). This observation challenges the well-established knowledge that *intI*1 sequence recovered within clinical settings has identical/nearly identical protein (≥98% protein identity) (24) and/nucleotide sequence (99%–100% nucleotide identity) (18) to each other. As such, this implies that *intI*1-like sequences can also be present within clinical settings and not just restricted to environmental settings as originally thought.

Our sequence results also highlight that the primer sets show that it was not possible to distinguish between *intI*1 and the lesser conserved *intI*1 variants (*intI*1-like, <98% protein similarity) that have been shown to coexist within these settings and similar environments (8, 18). These less conserved CL1-integron integrases (*intI*1-like) have been found, for example, on the chromosome of non-pathogenic *Betaproteobacteria* isolated from biofilms and soil, and the entrained gene cassettes encoded other functions rather than AMR (19). *intI*1-like may therefore not contribute to AMR but will contribute to *intI*1 Q-PCR signal. None of the primers, not even with the addition of a TaqMan or MGB probe, were able to distinguish between *intI*1 and *intI*1*-*like. As such, quantified *intI*1 gene abundance could potentially be overestimated. However, designing new primers over longer region but still suitable for Q-PCR, capable of distinguishing both variants can be a challenge. This is because the *intI*1-like protein sequence identity between bacteria species can vary when compared to the reference *intI*1 *P. aeruginosa* reference sequence (pVS1, AAA25857.1). For example, the *IntI*1-like protein sequence from *Salmonella enterica subsp* (KMJ40944.1), a gamma-proteobacteria and *Comamonas thiooxydans* beta-proteobacteria (WP_012838479.1) shared 87.8% and 92.8% identity to the reference pVS1 *IntI*1 protein sequence, respectively. As such, the varying conserved region shared between the *IntI*1, and *IntI*1-like protein sequence variant makes it a challenge to design a primer that exclusively distinguish both variants.

The potential contributions of *intI*1-like abundance to the overall abundance of *intI*1 gene quantified *via* Q-PCR suggest that *intI*1 abundance may not be an adequate or reliable proxy for inferring overall AMR abundance. Therefore, other potential proxies such as the *qacEΔ*1 (confer antiseptic resistance) or *VanA* (confer vancomycin resistance) (52) should be investigated for reliable estimation of overall AMR abundance in polluted environments.

This work has also shown the impact of using different primers on the interpretation of the findings and, in turn, our understanding of the risk of AMR. While across our septic tanks, the three best-performing primer sets revealed the same overall trends (Fig. 2), they did on occasion change the statistical difference between samples. For example, there were statistically higher *intI*1 gene abundances in the effluent than the sludge of the CST-healthcare unit when quantified with one (DF-DR) of the three primer sets but no difference when using the other two (F3-R3 and F7-R7) (Fig. 2). Depending on the primer set used, our understanding of the role of wastewater in the dissemination of CL1-integron and entrained AMR gene to the wider environment differed, highlighting the need for primers standardization whether comparisons and environmental meaning are to be gained from the large body of literature and work currently being undertaken in this area. With this in mind, from the work carried out validating and comparing the primer sets, we arrived at three very good primer sets, albeit with the lack of specificity for *intI*1. As the addition of the TaqMan and MGB probes did not offer increased specificity, we recommend F3-R3 primer set and SYBR green assay (28). This primer set has previously been extensively used in the literature to survey CL1-integrons from a wide-ranging environment (49) and here we have further demonstrated their suitability to quantify mRNA also. For this, a gene-specific RT-Q-PCR assay performed best as previously demonstrated (50).

The combination of quantitative PCR and amplicon sequencing approach offers a rapidly targeted and cost-effective alternative, in contrast to shotgun metagenomics. This approach permits reliable and accurate profiling of functional genes from various environment, and is, therefore, highly recommended in future studies.

### Ecological risk assessment of septic tanks in contributing to *intI*1 gene abundance to the environment

Comparing the abundance of *intI*1 genes (copies/ng DNA) among the different septic tanks, we showed that they were higher in the effluent compared to sludge, for all three reactors (CST-household, CST-healthcare, and SST-household), irrespective of the *intI*1 primer set used, with the highest gene abundance quantified in the conventional healthcare (CST-healthcare) effluent (Fig. 2). This finding was consistent with a previous study that reported higher *intI*1 relative gene abundance (normalized abundance to the *16S rRNA* copies) in hospital effluent compared to urban or municipal WWTP effluent (48). Healthcare institutions are among the primary consumers of antimicrobials particularly antibiotics (48). As such, stronger selective pressures are imposed within the bacteria communities, which, in turn, drives the acquisition of resistance genes carried within key vectors such as CL1-integron, to ensure their survival from the constant threat of antimicrobials within theWWT system.

Of the three reactors, lower *intI*1 gene abundance per ng of DNA was quantified in the household solar septic tank (SST) samples (sludge and effluent) compared to the conventional tanks (CST-healthcare and CST-household) by two (DF-DR and F7-R7) of the primer sets while the third (F3-R3) only quantified lower *intI*1 gene copies in the SST-household effluent and not the sludge sample (Fig. 2). Nonetheless, this implies that the increased temperature potentially plays a role in reducing CL1-integron from WWT and thus, the abundance entering the environment. This finding agrees with our proposed hypothesis of decreased *intI*1 gene abundance as a result of increased temperature driving enhanced wastewater treatment. Although the target internal temperature (50°C–60°C) within the solar tank was not consistently achieved, our finding is consistent with the recent study by Zhang and colleagues (53), who investigated removal of CL1-integron and entrained AMR genes from anaerobic digestors operated at higher (thermophilic: 55°C) and lower (mesophilic: 35°C and 25°C) temperatures and reported statistically lower *intI*1 gene abundance and removal at higher temperature. In addition, statistically lower *16S rRNA* gene abundance was reported at the higher temperature, coupled with a lower relative abundance of AMR gene cassettes, albeit slightly higher ARG subtypes were detected with the higher temperature.

Although typified by poor treatment performance (7), the conventional household tank was able to reduce *intI*1 gene abundance in the effluent from the influent by 31.21% to 42.33%, depending on the *intI*1 primer set used. This finding is consistent with a previous study by Chen and Zhang (13), although Chen and co-worker reported higher *intI*1 removal in the effluent from the influent (estimated around 1.9 to 2.3-log removal) for two of the three onsite domestic WWT associated with single-family usage investigated than observed in this study. In the third onsite, domestic WWT associated with single-family use enrichment of *intI*1 gene abundance was reported. However, the better removal from the two tanks in their study (13) may be due to the additional secondary treatment incorporated into the tank, such as eco-filter, constructed wetland, and multi-soil layering, prior to discharge to the environment which was not done in our study.

WWT sludge represents an additional source of CL1-integron and entrained AMR genes to the environment, particularly if improperly managed (i.e. improperly disposed of without further treatment), which further exacerbates the global AMR burden (54). In the Global south regions such as Thailand and Vietnam, only 10%–20% of the fecal sludge generated are estimated to be adequately disposed of, while the vast majority are discharged directly to the environment (54). With the high *intI*1 abundance quantified in the sludge for the three reactors, coupled to the already high abundance in the effluent, we found that, on average, 1.22x10^5^ to 1.48 × 10^5^, 8.41 × 10^4^ to 1.1 × 10^5^, and 7.73x10^4^ to 9.4 × 10^4^ *intI*1 gene copies per ng DNA (depending on primer set), enters the environment via the CST-household, CST-healthcare, and SST-household, respectively. This is significant when taking into account the proportion of global population (2.7 billion people) estimated to be served by onsite decentralized WWT including septic tanks (55). Thus, highlighting septic tanks as an important source of CL1 to the environment, further supports the broader knowledge that WWT in general, is a major source of CL1-integrons and entrained resistance genes to the environment. For the CST-household tank with accessible influent sample, while the load of *int*I1 was decreased from the influent, the abundance of *intI*1 quantified in the sludge and effluent by the different primer sets represent a significant source of *intI*1to the environment and therefore, emphasizes the need to optimize the conventional septic tank for AMR removal.

The increased abundance of CL1-intgerons entering the natural environment from WWT coupled with a slow decay rate [*intI*1 halve-life estimated ≥ 1 month in soil (56)] increases the risk of acquisition and dissemination into broader bacteria taxa, especially clinically relevant human pathogenic bacteria including *Acinetobacter baumannii* (57), *Proteus mirabilis* (58, 59), and *P. aeruginosa* (60, 61).

### Conclusions

This present study has provided insight into the importance of primer choice especially in the context of validating the *intI*1 as a suitable proxy for AMR pollution, and the need for standardization across studies to comprehensively understand the role in which wastewater plays in disseminating CL1-intgerons and by extension AMR genes to the environment. Further work is needed to determine whether the *intI*1 is indeed a suitable proxy for overall AMR gene abundances.

Moreover, we showed septic tank decentralized wastewater, particularly the conventional healthcare tank (CST-healthcare), can be a significant source of CL1 integron to the environment *via* the effluent and sludge if the sludge is directly applied to the environment without undergoing additional treatments, such as wetlands, to reduce the *intI*1 gene load. Thus, supports growing evidence that WWT in general is a major source of CL1-intgerons and associated resistance genes to the wider environment which further exacerbates the global burden from AMR.

## Data Availability

The sequence data for this study have been deposited in the European Nucleotide Archive (ENA) at EMBL-EBI under accession number PRJEB65102 with the meta data provided in the supplementary material 2.

## SUPPLEMENTAL MATERIAL

Supplementary Material 1 aem.01071-23-s0001.docx Supplementary Material 1.

Supplementary Material 2 aem.01071-23-s0002.xlsx Meta-data for submitted MiSeq sequence data.

ASM does not own the copyrights to Supplemental Material that may be linked to, or accessed through, an article. The authors have granted ASM a non-exclusive, world-wide license to publish the Supplemental Material files. Please contact the corresponding author directly for reuse.
